# Acoustic Dual-Function Communication and Echo-Location in Inaudible Band

**DOI:** 10.3390/s22031284

**Published:** 2022-02-08

**Authors:** Gabriele Allegro, Alessio Fascista, Angelo Coluccia

**Affiliations:** Department of Engineering, University of Salento, Via Monteroni, 73100 Lecce, Italy; gabriele.allegro@studenti.unisalento.it (G.A.); alessio.fascista@unisalento.it (A.F.)

**Keywords:** joint communication and ranging, acoustic communications and sensing, inaudible communications, localization

## Abstract

Acoustic communications are experiencing renewed interest as alternative solutions to traditional RF communications, not only in RF-denied environments (such as underwater) but also in areas where the electromagnetic (EM) spectrum is heavily shared among several wireless systems. By introducing additional dedicated channels, independent from the EM ones, acoustic systems can be used to ensure the continuity of some critical services such as communication, localization, detection, and sensing. In this paper, we design and implement a novel acoustic system that uses only low-cost off-the-shelf hardware and the transmission of a single, suitably designed signal in the inaudible band (18–22 kHz) to perform integrated sensing (ranging) and communication. The experimental testbed consists of a common home speaker transmitting acoustic signals to a smartphone, which receives them through the integrated microphone, and of an additional receiver exploiting the same signals to estimate distance information from a physical obstacle in the environment. The performance of the proposed dual-function system in terms of noise, data rate, and accuracy in distance estimation is experimentally evaluated in a real operational environment.

## 1. Introduction

Acoustic signals are of interest in the telecommunications industry for various remote applications, such as sonar for underwater civilian/military scenarios [1,2,3], modems for both aerial and underwater communications [4,5,6], as well as echography and other medical applications involving image reconstruction [7,8,9]. Networks of acoustic sensors, called wireless acoustic sensor networks (WASNs), are increasingly adopted to support sensing and monitoring applications in the emerging paradigm of the smart cities. Prominent examples include the monitoring of urban noise [10,11], the deployment of advanced acoustic surveillance systems [12], and more generally indoor and outdoor applications benefiting from the extraction of contextual information (e.g., proximity, ranging) from acoustic communications [13]. In general, acoustic signals have several possible applications in relation to the adopted frequency range: the frequency band audible to humans (audio band), the frequency band higher than the audible ones (ultrasonic band), or the frequencies lower than those audible (infrasonic band). The audio band is used for natural acoustic communications for humans and animals and in short-range telecommunications system, the ultrasonic band (typical of bats and dolphins) is used for imaging applications, while the infrasonic band (present in elephants) is adopted for earthquake detection.

Acoustic communications, which as mentioned found major applications in underwater as well as wired contexts, are recently experiencing renewed interest in some wireless scenarios. In fact, the use of the electromagnetic (EM) spectrum is increasingly saturated for commercial, social, and military applications, through the use of Wi-Fi modems, GPS, smartphones, and Internet of Things (IoT) devices [14,15,16,17,18]. It is thus useful to introduce a different communication channel to guarantee the continuity of necessary services; a contribution to this problem is given by acoustic communications, which makes available a dedicated channel, independent from the EM one. Communication through the acoustic channel is possible for many off-the-shelf devices, such as smartphones, computers, TVs, etc., since they are equipped with transducers for the transmission and reception of acoustic signals, namely speakers and microphones. Through the same channel it is possible to perform ranging as in a sonar system. This is of particular interest for indoor localization, where access to satellite positioning systems such as GPS is denied [19]: it is possible to carry out localization tasks using the acoustic channel, with acoustic devices already present in many rooms (speakers and microphones) [20,21,22].

A very recent research direction is also to integrate some sensing functionality with communications. For EM signals, this is expected to be a key feature of the 6G cellular networks, allowing exploiting the dense cell infrastructure of 5G for constructing a perceptive network [23,24,25]. It is foreseeable, in fact, that future networks will go beyond classical communication and provide a sensing functionality to measure the surrounding environment [26,27]. This motivates the recent research theme of Integrated Sensing and Communications (ISAC) [28], which however concerns radiofrequency communications and radar systems. In fact, ISAC was first implemented over a missile range instrumentation radar via pulse interval modulation, where information was embedded into a group of radar pulses [29]. Such systems are defined by different names, e.g., joint radar and communication or dual-functional radar-communications [30]. The sensing functionality in these systems mainly refers to radar sensing since, as a major representative of sensing technologies, radar’s development has been profoundly affected by wireless communications, and vice versa [28].

Motivated by the above, we developed a system that allows performing dual-function communication and echo-location (DFCE) in inaudible band, with low-cost hardware. We present an acoustic waveform design that tries to strike a balance between communication and ranging performance, while fulfilling the challenging constraints of the inaudible band. Our work proposes an acoustic communication system between devices, such as smartphones and common speakers, which allows sending and receiving data. Through the same waveform that transports the information, the DFCE system can estimate distances according to the principles of acoustic echo-locator. To the best of the authors’ knowledge, this is the first demonstration of low-cost acoustic system in inaudible band able to perform joint communications and echo-location, and can represent a starting point for more advanced applications in different scenarios.

The remainder of the paper is organized as follows. In Section 2, we provide some background on acoustic communications and review the state-of-the-art approaches targeting the problem of acoustic localization and sensing. In Section 3, we present in detail the design of the proposed DFCE system, illustrate its main parameters, and discuss the fundamental trade-offs between communication and ranging. Then, in Section 4 we conduct experimental analyses in a real environment to assess the performance of the proposed DFCE system in terms of data rate and accuracy in distance estimation, using a testbed based on low-cost off-the-shelf hardware. We conclude the paper in Section 5.

## 2. Background and Related Work

Acoustic communications are attractive because they do not require extra hardware on either transmitter and receiver sides, which facilitates numerous near-field communications (NFC) use cases such as mobile payment and data sharing, very important tasks in IoT and other applications. The scientific literature explores acoustic communications separately from acoustic localization. Existing approaches focused on which band to use (audible, inaudible, ultrasound), and on the efficiency of the various acoustic modulations. Many of the works share some common signal processing steps, in particular for the sake of achieving high signal-to-noise ratio (SNR), since acoustic sensors (especially microphones) are quite sensitive and vulnerable to background noises, channel distortions, and multipath effects.

Noise generally arises from in-band and out-of-band interference. In common processing, out-of-band interference can be filtered out by means of digital filters such as finite impulse response. Another typical processing block is a matched filter, which seeks the waveform of interest in noise by correlating the measurements with a known reference signal. A common problem for inaudible transmission is that the audio speaker diaphragm inertia can cause *ringing* effects in the presence of rapid amplitude or phase variations in the transmitted signal: the speaker diaphragm inertia will generate out-of-band audible noise, due to the impulsive nature of the signal, despite the transmitted signal occupying inaudible frequency bands. To solve this problem, it is possible to introduce a smooth increase and decrease the amplitude of the first and last few samples (fade-in, fade-out) in the case of amplitude shift keying (ASK) modulation, and use a continuous-phase frequency shift keying (FSK) modulation to reduce impulsive noise.

In [31], multiple tones are used to transmit data at 5.6 kilobits per second (kbps) in an audible mode (735–410 Hz), or a single tone for 1.4 kbps rate in an inaudible mode (18.4 kHz); both modes work up to 2 m distance with line-of-sight (LOS) communication. In [32], an application is presented which implements orthogonal frequency division multiplexing (OFDM) with binary and quadrature phase shift keying (PSK) modulation schemes in the 6–7 kHz band. The proposed system aims at substituting NFC with a data rate of up to 800 bps in a very short range, less than 20 cm, and achieves a maximum data rate of 2.4 kbps. In [33], a communication system based on tone modulation is presented: it leverages the presence or absence of audible tone signals to embed information with ASK. This approach achieves a data rate of about 5 kbps with multiple audible tones, which reduces to 1.4 kbps when a single inaudible tone is applied. It reaches a maximum communication range of 2 m under LOS condition.

The authors in [34] proposed leveraging the *masking effect* of the human hearing system to achieve inaudible acoustic communication. This approach employs OFDM modulation and achieves 240 bps data rate. The masking effect happens when a signal is superimposed to another sound that is playing (a masker). The masker does not need to have the frequency components of the original signal for masking to happen, and a masked signal can be heard even though it is weaker than the masker. In [35], the authors focused on inaudible acoustic communication for off-the-shelf audio interfaces in long-range indoor environments, adopting a *chirp* signal, which is a signal in which the frequency varies linearly with time, increasing (up chirp) or decreasing (down chirp). By adopting such a signal, originally introduced in radar applications, the range is extended up to 25 m, to support most smart devices equipped with microphones and selective frequency responses. The authors developed a software digital modem for smartphones that can efficiently demodulate the chirp signal by combining fast Fourier transform and Hilbert transform, at a low data rate of approximately 16 bps. They choose the 19.5–22 kHz range and up/down chirp to identify bits, using the correlation function to match the received sound with up/down chirp, and find the maximum by smoothing the auto correlation curve, whose envelope is obtained via Hilbert transform.

In [36], a new form of real-time dual channel communication over speaker-microphone links, called Dolphin, was proposed. Dolphin generates composite audio for the speaker by multiplexing the original audio, for the human listener, and the embedded data signals, for smartphones. The composite audio can be rendered for human ears without affecting the content perception of the original audio. The user listens to the audio as usual without sensing the embedded data; in the meantime, the data signals carried by the composite audio can be captured and extracted by the smartphone microphones. The application belongs to the audible communication group, using masking effect. The authors adopted OFDM with on-off keying (OOK), a special form of ASK, in the bandwidth between 17 and 20 kHz. For the convenience of data transmission and decoding, they divide the embedding data-streams into packets. A packet consists of a preamble and 31 symbols, each preceded by a cyclic prefix. The preamble is used to synchronize the packet and the symbols contain data bits. At the receiver side, the system also exploits the channel estimation, to compensate for the effects of the channel. In [37], a classic cyclic redundancy check code to is added for error detection on the received data.

The system called Backdoor, proposed in [38], uses ultrasound signals that are naturally inaudible to humans yet completely recordable by all microphones without any hardware or software changes. However, specific hardware for the emission of ultrasound is required, which is more difficult to have in practice than a common speaker.

An acoustic-enabled mesh network was proposed in [39]. This approach leverages frequency hopping spread spectrum and achieves 20 bps at a distance up to 20 m. The systems works in the audible band, below 18 kHz. In particular, a sequence of known tones belonging to a single musical scale is used as the spreading sequence. The transmitter sends the information bits on a sequence of different frequencies; this sequence is known to the receiver which extract the data knowing the encryption key.

A complete and functional commercial application in inaudible band, whose development started as a university spin-off in 2011, is called Chirp [40]. It uses FSK for its modulation scheme, due to its robustness to the multipath propagation present in real-world acoustics, compared to schemes such as PSK or ASK. For spectral efficiency, Chirp uses an *M*-ary FSK scheme, encoding input symbols as one of *M* unique frequencies. Each symbol is modulated by an amplitude envelope to prevent discontinuities, with a guard interval between symbols to reduce the impact of reflections and reverberation at the decoding stage. Chirp has been recently acquired by Sonos (a leading innovative loudspeaker brand), then its software development kit, public and open source until March 2020, is not available anymore—hence the details of the implementation of the current version are not disclosed (Chirp holds 14 data-over-sound related patents (4 granted) dating back to 2010, covering custom modulation scheme, de-reverberation, noise reduction, and multi-environment optimization. According to the company, this would allow data to be transmitted using sound in the harshest environments.).

For acoustic ranging, authors in [41] develops a basic echo-locator to calculate distance with sound sensors. A microphone and the internal speaker of the smartphone are used to this aim: after transmitting the signal and recording the reflection, the cross-correlation (after filtering) is used to compare the received signal with the original pulse. This method implements a transmission of a signal for which the identification of the maximum value of the cross-correlation function is less ambiguous, i.e., a linear chirp.

BeepBeep [42] is the first work that uses acoustic signal for precise ranging on commodity mobile devices. It calculates the distance between pair-wise transceivers by estimating the propagated time of acoustic signals. BeepBeep avoids tight synchronization via a two-way sensing approach: one device first emits a chirp signal, then upon detection, the other device waits for an arbitrary period and then emits another chirp signal. Both transceivers finally calculate the time difference between the events of transmission and reception locally by counting the number of acoustic samples. A central server is used to compute the results from the time differences.

BatMapper [43] realized a radar using acoustic signals (sonar) on off-the-shelf mobile devices for indoor floor map construction. The acoustic ranging module in the application consists of sound emission, sound recording by two microphones in a single device, and a series of signal processing steps to produce distance/amplitude measurements for echo candidates in both microphones. BatMapper also leverages three sensing modalities: acoustic, gyroscope, and accelerometer for fast, accurate floor plan construction. The user walks along corridors and inside rooms while holding the phone. The phone keeps emitting and recording sound signals, detects sound reflections and measures their distances by using amplitudes. Two frequencies are employed due to the hardware limitations and heterogeneous properties of the smartphone’s two microphones (16 kHz for top microphone and 10 kHz for bottom microphone) and also because for the same emitted energy, low frequency sounds propagate farther than high frequency ones.

In the present work, we developed a system that allows simultaneously performing communication and ranging operations, in inaudible band, with low-cost hardware. Our acoustic waveform design tries to strike a balance between communications and ranging performance, while fulfilling the constraints of the inaudible band. Our work in particular proposes an acoustic communication system between devices, such as smartphones and common speakers, which allows sending and receiving data but, at the same time, can estimate distances according to the principles of acoustic echo-locator, by implementing a DFCE system. To the best of the authors’ knowledge, this is the first demonstration of such an acoustic system in inaudible band, and can represent a starting point for more advanced applications in different scenarios.

## 3. Design of Dual-Function Communication and Echo-Location System

**Figure 1:**
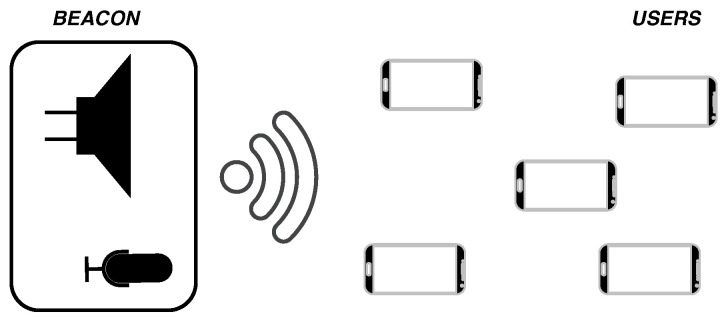
Reference scenario of the proposed DFCE system: broadcast beacon-to-users communication paradigm.

This section presents the design of the dual-function system. The designed waveform in inaudible band, for both communication and ranging purposes, is discussed together with the associated processing algorithms. The reference scenario for the DFCE system is broadcast, with a single node (beacon) communicating with multiple devices (users), as shown in Figure 1. Three sub-phases can be identified in a typical processing task. First, the beacon broadcasts a signal, with informative content; second, the signal travels in the channel and is received by the users; third, the beacon receives the natural echoes caused by the reflections of the transmitted signal with the physical devices. Thus, the beacon can estimate the distances based on the received echoes, while the users can decode the information in the signal transmitted by the beacon.

### 3.1. Transmission and Ranging

One of the main challenges of using the inaudible band is, by its nature, the limited extent of the available bandwidth; this is related to the inability of the human ear to hear acoustic phenomena beyond 20 kHz, but this value progressively decreases with age. Since, however, off-the-shelf microphones have good response until 22 kHz, there is a margin to be exploited for inaudible communications. While the upper frequency limit, denoted by $f_{H}$, cannot exceed 22 kHz, the lower frequency limit $f_{L}$ is somewhat dependent on age. If one knows that the users involved in the application are of a certain age, this number can be chosen in a more tailored way; more often, however, it is reasonable to expect that age of involved people may span several years, so a conservative value is advisable. In the design of our DFCE system, we aim at preserving acoustic signals that are not audible to the human ear, but sensitive to common microphones, and then choose the operating frequency range between $f_{L}=18$ kHz and $f_{H}=22$ kHz. The maximum available bandwidth $f_{H}-f_{L}$ is therefore only 4 kHz. In this region, as discussed in [31], it is possible to reach a maximum rate of 1.4 kbps with an FSK modulation. FSK modulation allows one to have a higher rate in the inaudible band compared to all the other modulations. However, our system must not only establish efficient communication, but should be able to estimate the distance with an acceptable relative error, which poses additional constraints on the waveform design.

The basic principle of echo-location is to exploit the transmission of a pulse for distance estimation. Such a pulse travels through the air until it encounters an object; at that point it is reflected back, i.e., the reflected waves travel back to the transmitter which records the echo by means of a microphone. This signal is compared with the transmitted pulse, typically by means of the cross-correlation function. For two signals f(t) and g(t), the cross-correlation is defined as [44]

(1) $$R\left(\tau \right)=\int_{-\infty }^{+\infty }f^{*}\left(t\right)g\left(t-\tau \right)dt$$

where ${}^{*}$ denotes the complex conjugate. When the comparison between the received signal and the transmit template matches for some delay (lag) τ, the result of the cross-correlation function will be maximum. It is known that, for accurate distance estimation, the use of a pseudo-noise random code (direct sequence spread spectrum, DSSS) or a chirp pulse is much more convenient than a single tone of a FSK modulation. Therefore, there are two conflicting objectives in the dual communication-ranging task.

**Figure 2:**
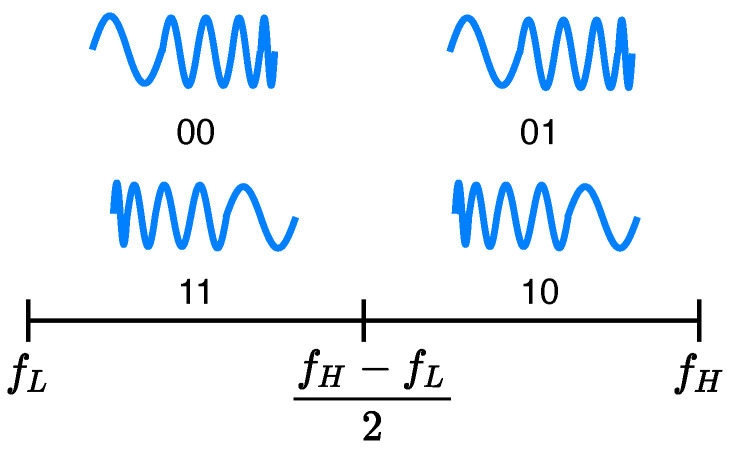
Up-down chirps used in each sub-band to encode four different symbols (quaternary modulation).

To carry information content through the waveform, while retaining good ranging capabilities, we adopt a chirp slope keying modulation. The basic idea is to transmit frequency modulated pulses in which the frequency changes continuously, increasing or decreasing, for the duration of the pulse. However, the waveform and bandwidth constraints discussed above also need to be considered. We observe that two different signals are possible in 4 kHz using two chirps with up/down slopes, in the available inaudible band between *B*, with continuous band of B/2, respectively, between $f_{L}$ up to $(f_{H}-f_{L})/2$ and $(f_{H}-f_{L})/2$ up to $f_{H}$. Up/down chirps can be used in each sub-band to identify two different symbols. In this way it is possible to encode two bits of information by means of four distinct signals, i.e., four symbols (quaternary modulation). Decreasing the value of the frequency sweep range of the chirp reduces the accuracy of range estimation, but it increases the data rate, and vice versa. In the diagram in Figure 2 we can see a representation of the four chirp-based signals, their polarity, and the occupied band.

The up/down chirp signals, in the respective useful bands, are [45]

(2) $$f_{\alpha }\left(t\right)=\frac{1}{\sqrt{T}}\mathrm{rect}\left(\frac{t}{T}\right)e^{j2\pi f_{0}t+j2\pi \alpha t^{2}}$$

(3) $$f_{-\alpha }\left(t\right)=\frac{1}{\sqrt{T}}\mathrm{rect}\left(\frac{t}{T}\right)e^{j2\pi f_{0}t-j2\pi \alpha t^{2}}$$

where *j* denotes the imaginary unit, $f_{0}$ is the carrier frequency, α=B/T is the chirp slope, *T* is the pulse width, *B* is the waveform bandwidth, and rect(t) the rectangular pulse defined as

(4) $$\mathrm{rect}\left(t\right)=\left\{\begin{array}{c} 1,\;|t|\leq 1/2\\ 0,\;|t|>1/2 \end{array}\right..$$

Chirps, even in the inaudible band, may generate low-volume noise produced by the ringing effect in the audio band, but less significant than all the other modulations (OOK, ASK, PSK, DSSS).

The signal broadcast by the beacon in the first phase, $s_{\mathrm{beacon}}\left(t\right)$, is thus chosen as a sequence $N_{c}\geq 1$ chirps, i.e.,

(5) $$s_{\mathrm{beacon}}\left(t\right)=\sum_{i=1}^{N_{c}}s_{i}\left(t-iT\right)$$

where each $s_{i}\left(t\right)$ is one of the four possible signals in the quaternary chirp slope modulation discussed above. The beacon then receives the echo signal from physical obstacles, possibly corresponding to user devices, with superimposed environmental noise n(t):

(6) $$s_{\mathrm{RX},\mathrm{beacon}}\left(t\right)=s_{\mathrm{echo}}\left(t\right)+n\left(t\right)$$

where $s_{\mathrm{echo}}\left(t\right)$ is a modified version of the transmitted chirp sequence $s_{\mathrm{beacon}}\left(t\right)$. Typically, $s_{\mathrm{echo}}\left(t\right)$ will contain a stronger component due to the LOS and possible replicas of the signal due to multipath reflections in the environment. Since in the considered scenario the bandwidth *B* is very narrow, pulses are sufficiently long compared to the delay spread of the channel, which means that the flat fading model can apply. As a consequence, we can consider $s_{\mathrm{echo}}\left(t\right)\approx A\left(t\right)s_{\mathrm{beacon}}\left(t\right)$ where A(t) accounts for the fading effects. Moreover, since we consider a slowly varying scenario, it is possible to approximate A(t) as a constant for the whole duration of $s_{\mathrm{beacon}}\left(t\right)$, i.e., A(t)≈A. The resulting model $s_{\mathrm{echo}}\left(t\right)\approx As_{\mathrm{beacon}}\left(t\right)$ is therefore the typical narrowband (flat and slow) fading, for which matched filtering can be adopted as effective detection approach.

Specifically, if a target is present at distance *d*, the application of the matched filter to the received signal yields the auto-correlation (or the inner product), therefore producing a peak that is associated to signal detection. In particular, the beacon detects the echo as the index corresponding to the maximum of the cross-correlation, computed on the sampled signals at rate $F_{s}=1/T_{s}$, where *H* is the number of samples of recorded sound [46]

(7) $$R_{\mathrm{RX},\mathrm{beacon}}\left(k\right)=\sum_{h=0}^{H-1}s_{\mathrm{RX},\mathrm{beacon}}^{*}\left(hT_{s}\right)\;s_{\mathrm{beacon}}\left(hT_{s}-kT_{s}\right).$$

Actually, if the beacon device records the audio continuously, two peaks will appear in $R_{\mathrm{RX},\mathrm{beacon}}\left(k\right)$: the first, stronger one, corresponds to the transmitted signal, the second, weaker one, corresponding to the echo signal from the target.

From the difference between these two time instants, converted in seconds by multiplying for the sampling time $T_{s}$, it is possible to estimate the *round trip time* (RTT) between the beacon and the target, and finally the distance estimate is

(8) $$\hat{d}=\frac{\mathrm{RTT}}{2}v$$

where v=340 m/s is the speed of the sound wave.

### 3.2. Detection and Demodulation

Users detect the broadcast signal by using again the cross-correlation function, which as seen is typically implemented in the digital domain by using the sampled version of $s_{\mathrm{beacon}}\left(t\right)$ at regular time instants $kT_{s}$, and the digitized version of the four template signals. We recall that the latter are $f_{\pm \alpha }\left(t\right)$ in the two halves of each 2-KHz sub-bands, for a total of four different configurations corresponding to the four symbols of the quaternary constellation. In other words, there are four preset chirps $s_{m}\left(t\right)$, m=1,…,4: up/down chirp in 18–20 kHz band and up/down chirp in the 20–22 kHz band.

**Figure 3:**
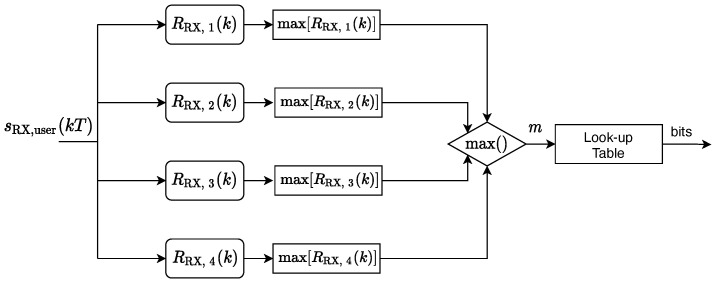
User demodulation scheme.

The signal received by the user, which can be written as

(9) $$s_{\mathrm{RX},\mathrm{user}}\left(t\right)=s_{\mathrm{beacon}}\left(t\right)+n\left(t\right)=A\sum_{i=1}^{N_{c}}s_{i}\left(t-iT\right)+n\left(t\right)$$

is stored and processed, by cross-correlating the recorded signal with each preset chirp, i.e.,

(10) $$R_{\mathrm{RX},m}\left(k\right)=\sum_{h=0}^{H-1}s_{\mathrm{RX},\mathrm{user}}^{*}\left(hT_{s}\right)\;s_{m}\left(hT_{s}-kT_{s}\right)$$

with m=1,…,4. In Figure 3, we present a diagram that associates the index of the signal identified with the bits to be received. The maximum among the maxima of the four cross-correlation results $R_{\mathrm{RX},m}\left(k\right)$ is selected; with reference to the look-up table in Table 1, this is a two-bit word. Each user then waits for the detection of a new transmission from the beacon, and repeats the process, storing the data in sequence and making them available at the application layer.

### 3.3. Trade-Off between Communication and Ranging

The proposed DFCE system is subject to the following trade-off between data rate and maximum measurable distance. After the beacon speaker transmits a signal, it must wait a certain period of time before transmitting the next signal, since during the waiting time it listens to the channel for a possible transmission echo. This is called *pulse repetition time* (PRT) and defines the maximum measurable distance. Other parameters play also a role, in particular, the minimum measurable distance, linked to the length time of the chirp pulse, and the bit rate. The main parameters can be calculated from these formulas:

(11) $$d_{min}=vT$$

(12) $$d_{max}=v\;\left(\mathrm{PRT}+T\right)$$

(13) $$\mathrm{BPS}=\frac{\mathrm{BPB}}{\mathrm{PRT}+T}$$

where BPB is the number of bits per beacon, depending on the number of transmitted pulses $s_{i}\left(t\right)$ in a single transmission.

Table 2 illustrates four system implementation possibilities. As it can be seen in the table, the duration time of the chirp transmission directly influences the minimum measurable distance and the number of bits that a single transmission encodes, and consequently the bit rate; the latter is inversely proportional to the sum of the total time between the duration of the chirp and the PRT. The PRT in particular is directly proportional to the maximum measurable distance. In general, therefore, increasing the maximum measurable distance decreases the bit rate and vice versa. In the case of a chirp of 1 ms, the waveform encodes a single pair of bits, while in the case of chirp lasting 2 ms, the waveform is the sum of two chirps, each of which encoding two bits; as a result, four bits can be carried with a single transmission. It can be useful to choose the trade-off required for an application, for example one can sustain short-range communications at about 3 m with twice the bit rate compared to a system with extended communication range of about 7 m.

## 4. Experimental Performance Assessment of the Proposed DFCE System

**Figure 4:**
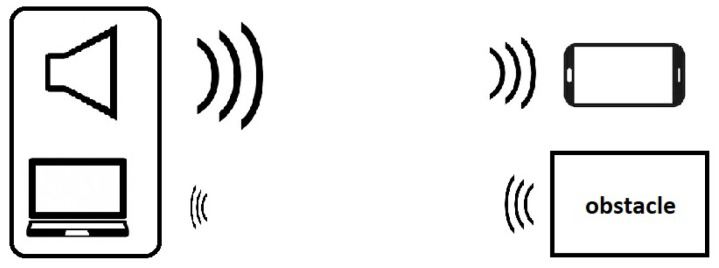
Experimental setup for acoustic dual-function communication and echo-location.

An experimental testbed was implemented to assess the performance of the proposed system, following the scheme reported in Figure 4. The software part was developed in Matlab, while the hardware part includes the following:

- A beacon, consisting of a standalone loudspeaker as acoustic transmitter and a laptop with integrated microphone as acoustic receiver. It is used for sending broadcast data and receive the echo signal.
- An off-the-shelf device, in particular a smartphone with an onboard microphone. This implements the receiver of the broadcast beacon signal.

**Figure 5:**
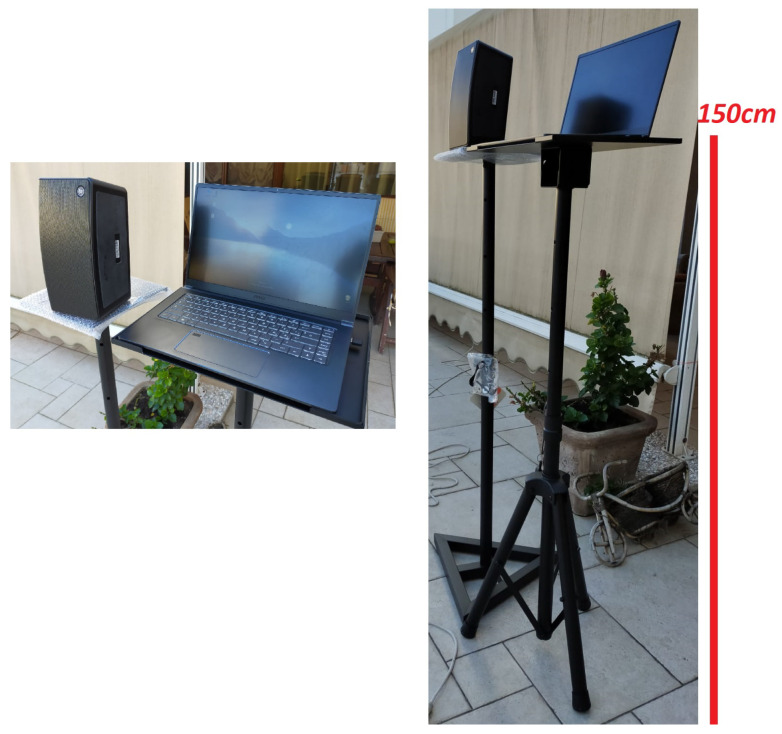
Beacon set-up with laptop integrated microphone and external loud-speaker.

**Figure 6:**
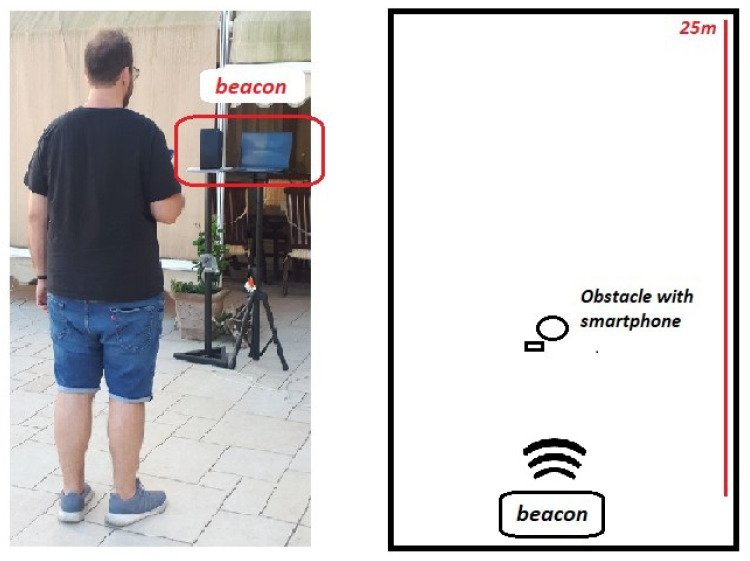
Set-up of the experimental testbed and test environment.

In particular, the setup shown in Figure 5 was adopted. It includes a 20 Watts-RMS/40 Watts-peak loudspeaker (model Mackie Free-play Home), with two full-range drivers with 2.5 in/64 mm and two passive radiators for extended bass frequencies with quasi-flat −3 dB response between 0.12 and 20 kHz. The beacon consists of a notebook connected to the external speaker via mini-jack cable. The system is positioned on two supports at a height of 150 cm, so that the position of the speaker and the microphone of the PC are aligned. The user receiver is an Android smartphone (OPPO A72), whose audio specifications are similar to most current smart devices, namely ADC with sampling frequency of 44.1 kHz, meaning that the maximum non-aliased frequency is 22.05 kHz. The smartphone is carried by one of the authors which is therefore the main obstacle reflecting the sound waves as shown in Figure 6. In general, significant echoes are generated by particularly reflective, non-porous material, with size comparable to 10–15 times the half wavelength of the frequencies emitted by the pulse. The smartphone/obstacle was positioned at several distances, so as to test the DFCE system under different operational conditions.

The environment in which the experiments were carried out is an open space with a perimeter of about 15 × 25 m covered by walls, without obstacles along the LOS between beacon and target. The measurements were repeated numerous times to ensure statistical consistency. The weather was clear, without rain and wind; the factors of humidity and temperature, which make a minimal contribution, were not taken into consideration. The levels in dB(Z) and dB(A) of the sound pressure level (SPL) were measured with a European-law class-1 sound level meter, calibrated in 2019 with ACCREDIA certification.

To evaluate the operating efficiency of the proposed DFCE system, various measurements of different parameters were performed:

- the SNR as a function of distance, to evaluate the maximum operating range; the audio noise produced by the modulation was also evaluated;
- the bit error rate (BER) as a function of the distances, to assess the communication performance;
- the error on the distance estimation to assess the echo-location performance.

**Figure 7:**
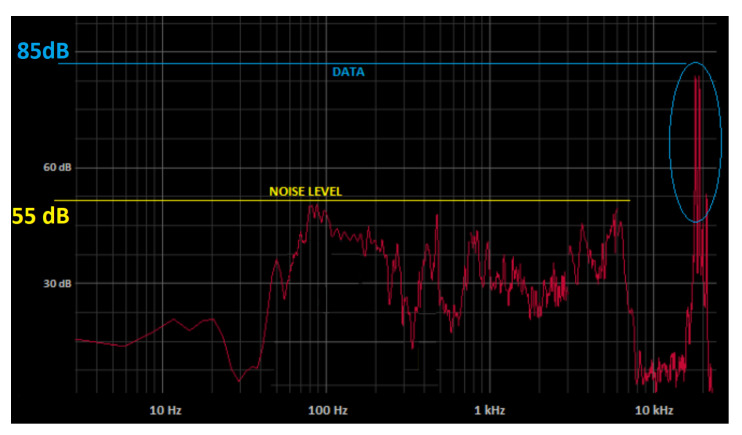
Sound Spectrum of the environment with transmitted signal in the inaudible band, and comparison with the average noise level.

In the considered outdoor environment, an acoustical background noise of about 55 dB(Z) SPL was measured on average; a value of 85 dB(Z) SPL was subsequently measured during the transmission of data pulses, where dB(Z) denotes a flat frequency response (no weighting). The noise measurement level in Figure 7, highlighted in yellow, is thus overall 30 dB less than the pressure level of the transmitted signal, highlighted in blue. However, if A-weighting based on isophonic curves for the human ear is considered (IEC 61,672:2003 standard), the SPL values become about 55 dB(A) and 65 dB(A) for background noise and transmitted data pulses, respectively, (regardless of age). This means that because the human ear is most sensitive to sound frequencies between 500 Hz and 6 kHz, the additional acoustic pressure generated by the transmitted signal is practically perceived as environmental noise. In general, we can see that the background noise is mainly concentrated in the band under 8 kHz, and is otherwise very small over 10–15 kHz. The inaudible system is thus intrinsically robust against background noise.

The SNR value can be expressed as a function of the transmission loss (TL), consequently distance *d*, source level (SL) and noise level (NL), as follows:

(14) SNR(d)=SL−NL−TL(d)=30−20logd[dB].

**Figure 8:**
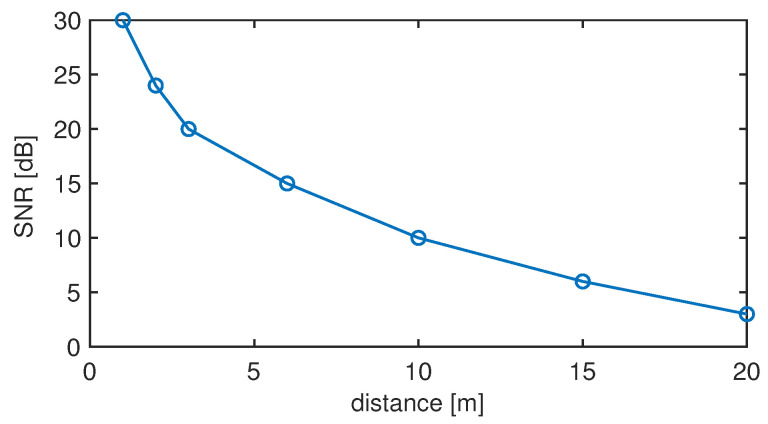
SNR as a function of the distance.

In Figure 8, we report the evolution of the SNR as a function of the distance. As it can be noticed, the system experiences an SNR of about 30 dB at short distances; as the distance increases up to 8–10 m, the SNR decreases accordingly until reaching a value of about 9–10 dB. It is only when the distance grows up to 20 m that SNR values fall below 5 dB.

To evaluate the transmission performance, we considered the BER, i.e., the number of errors on the decoded bits per unit of time. It provides a measure of the quality of the entire communication system, and can be calculated as the ratio between the number of erroneous bits and the number correctly decoded bits:

(15) $$\mathrm{BER}=\frac{N_{\mathrm{error}}}{N_{\mathrm{correct}}}.$$

**Figure 9:**
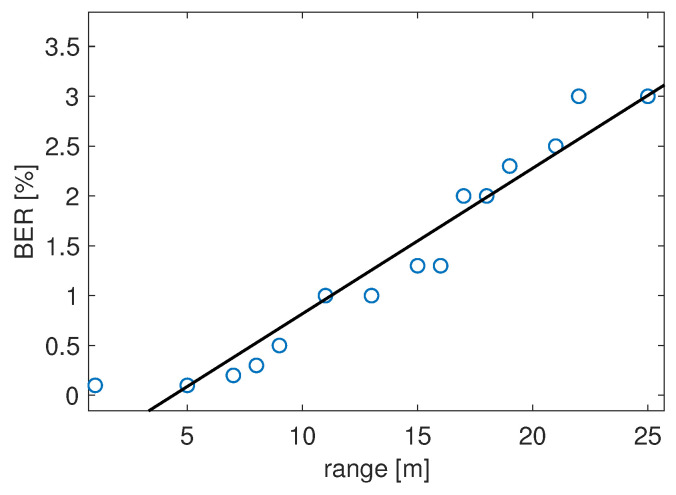
Communication performance in terms of BER as a function of the distance.

In our tests, the smartphone (receiving device) was placed at different distances from the speaker of the beacon, in the range of 1–25 m (a value in line with the state-of-the-art, as discussed in Section 2). The signal was transmitted at the rate of 10 bps, with a single pulse of 1 ms and a PRT set to 9 ms (for the sake of accurate ranging, as discussed later), for $10^{4}$ bits. The results, as a function of the distance, are shown in Figure 9. It can be seen that the BER is very small at short ranges, still quite small below 10 m, and grows above 2% between approximately 20 and 25 m.

More specifically, the BER is about 0.02% up to 6 m, less than 1% up to 10 m. This confirms that the communication with the smartphone is sufficiently reliable and the behavior of the BER is sufficiently predictable by means of a linear law.

To assess the echo-location performance of the waveform transmitted for communication, again various measurements were made and averaged, at several distances. The minimum measurable distance using a chirp with duration 1 ms, with the speed of sound of 340 m/s at room temperature is

(16) $$d_{min}=vT=0.34\;\left[m\right].$$

Indeed, the DFCE system is “blind” to targets that are too close in range; in particular, it is not possible to estimate distances *d* corresponding to delays shorter than the duration *T* of the probing signal. In other words, the transmission of the chirp must end before any possible echo can be detected, hence the minimum resolvable distance value in Equation (16) follows.

The system trade-offs allow measuring a maximum distance related to a total transmission time of T+PRT=20 ms (see Table 2) as

(17) $$d_{max}=v\left(T+\mathrm{PRT}\right)=6.8\;\left[m\right].$$

**Figure 10:**
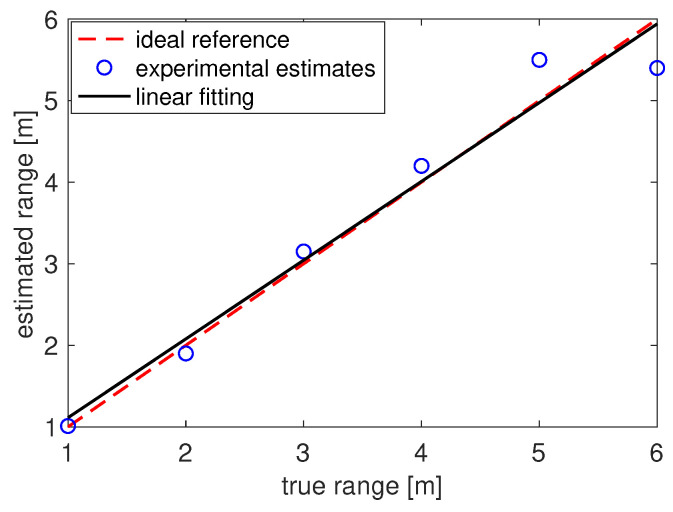
Echo-location performance in terms of estimated distance vs. actual distance.

Figure 10 shows the estimated distance $\hat{d}$ as function of the true distance *d*. The dashed red curve shows the ideal behavior with zero error, while the solid black curve shows the linear fitting computed using the values of the estimated distances (blue circles) obtained during the tests. The very good adherence between the reference and the estimated values reveals that the proposed DFCE system delivers accurate range estimates.

**Figure 11:**
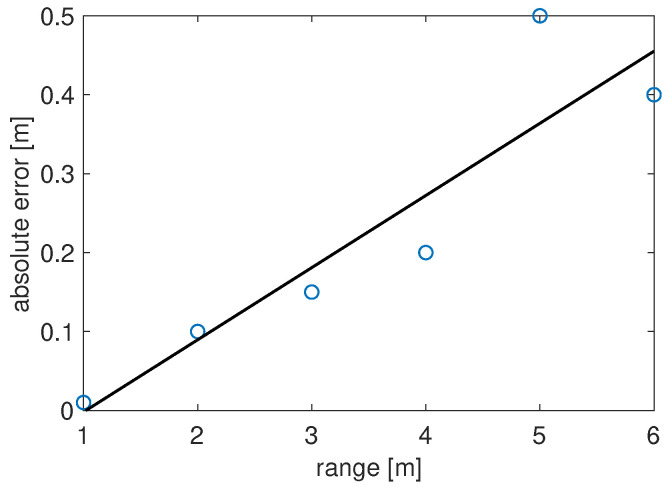
Absolute error as a function of the distance.

Finally, the absolute error was also evaluated, i.e.,

(18) $$E=|\hat{d}-d|$$

to better quantify the achieved accuracy. The graph in Figure 11 shows the results for a range varying between 1 and 6 m. As it can be noticed, the absolute error increases with the distance: it is a few centimeters for the short-range echo-location and it is about half a meter for distances between 5 and 6 m.

For longer distances, the performance starts to degrade. To corroborate the results, we performed several tests under different high noise environments. In particular, we performed worst-case experiments by introducing a source of white noise in the acoustic environment, which thus affects both the audible and inaudible bands. Results show that the proposed DFCE system is robust to severe environmental noise (even 80 dB) without any appreciable deviation in the measured error, provided that the signal stands out (even slightly) from the noise level, i.e., the SNR is above 0 dB. For environments where signal and noise are comparable in power (around 0 dB SNR) the estimator becomes imprecise, since the signal is completely buried below the noise level.

## 5. Conclusions

The paper investigated the feasibility of a “Dual-Function” system for acoustic ranging and communication in inaudible band, using off-the-shelf devices. While dual-function transceivers are more typical of RF systems, interest in acoustic solutions as an alternative to RF has recently increased. This is not limited to electromagnetic-denied scenarios but is also motivated by the appeal of having an additional dedicated channel available, independent of RF, to ensure continuity of some critical services, especially in areas where the EM spectrum may be heavily congested.

Our design of the DFCE system focused on the inaudible band, in particular the 18–22 kHz band, where microphone transducers are still sensitive to sound waves while the human ear is not. A suitable waveform was selected for signal modulation, which at the same time can be effectively used for echo-location; in particular, a chirp modulation was considered, where a sequence of pulses with different slopes (up chirp and down chirp) encode one bit and two frequency subbands encode a second bit, so producing a quaternary digital constellation. This allows the system to transmit data while exhibiting suitable cross-correlation properties to extract the time of flight information from the received echoes, so obtaining a range estimate.

The proposed solution was tested in an outdoor environment to derive the performance of the joint functions. Key performance metrics such as BER, maximum range vs. SNR, and ranging accuracy were computed in several experiments. Results reveal that the echo-locator can guarantee low absolute errors in a range between approximately 1 and 6 m, and in the same interval the communication performance can yield a BER as low as 0.02%. This shows that even the low-cost DFCE prototype built on the basis of the proposed design can work in practice with satisfactory BER and ranging error. Finally, it emerged that the noise produced by transmitting such acoustic signals in the audible band is slightly perceptible, intermittently, but only at a short distance from the source and in LOS conditions. Therefore, it is very tolerable and often inaudible to the human ear in most practical situations. Possible directions of future work can include the improvement of the communication and ranging performance of the proposed DFCE system, so as to make it applicable also in more complex environments. Another interesting direction that can be investigated is the extension of the DFCE system functionalities in multi-user scenarios. For these contexts, suitable mechanisms need to be devised in order to manage in particular the multiple access channel.

## Figures and Tables

**Table 1:** Association between chirp index and corresponding information bits.

Index *m*	Waveform	Bits
1	18–20 kHz up chirp	00
2	20–22 kHz up chirp	01
3	18–20 kHz down chirp	11
4	20–22 kHz down chirp	10

**Table 2:** Four possible configurations of DFCE system parameters.

Chirp Duration *T*	Min Distance	Max Distance	PRT	BPB	BPS
1 ms	34 cm	3.4 m	9 ms	2	20
1 ms	34 cm	6.8 m	19 ms	2	10
2 ms	68 cm	3.4 m	8 ms	4	40
2 ms	68 cm	6.8 m	18 ms	4	20

## Data Availability

The data presented in this study are available upon request from the corresponding author.

## Abbreviations

The following acronyms are used in this manuscript:

ASK	Amplitude Shift Keying
BER	Bit Error Rate
BPB	Bits per Beacon
BPS	Bits per Second
DFCE	Dual-function Communication and Echo-location
DSSS	Direct Sequence Spread Spectrum
EM	Electromagnetic
FSK	Frequency Shift Keying
IoT	Internet-of-Things
ISAC	Integrated Sensing and Communications
LOS	Line-of-Sight
NFC	Near-field Communications
NL	Noise Level
OFDM	Orthogonal Frequency Division Multiplexing
OOK	On-Off Keying
PRT	Pulse Repetition Time
PSK	Phase Shift Keying
RF	Radio-Frequency
RTT	Round Trip Time
SL	Source Level
SNR	Signal-to-Noise-Ratio
SPL	Sound Pressure Level
TL	Transmission Loss
WASN	Wireless Acoustic Sensor Networks
